# Targeted mutagenesis in wheat microspores using CRISPR/Cas9

**DOI:** 10.1038/s41598-018-24690-8

**Published:** 2018-04-25

**Authors:** Pankaj Bhowmik, Evan Ellison, Brittany Polley, Venkatesh Bollina, Manoj Kulkarni, Kaveh Ghanbarnia, Halim Song, Caixia Gao, Daniel F. Voytas, Sateesh Kagale

**Affiliations:** 1 Canadian Wheat Improvement Flagship Program, National Research Council Canada, 110 Gymnasium Place, Saskatoon, SK S7N 0W9 Canada; 2 Department of Genetics, Cell Biology, and Development, Center for Genome Engineering, University of Minnesota, Saint Paul, MN 55108 USA; 3 State Key Laboratory of Plant Cell and Chromosome Engineering, Center for Genome Editing, Institute of Genetics and Developmental Biology, Chinese Academy of Sciences, Beijing, 100101 China

## Abstract

CRISPR/Cas9 genome editing is a transformative technology that will facilitate the development of crops to meet future demands. However, application of gene editing is hindered by the long life cycle of many crop species and because desired genotypes generally require multiple generations to achieve. Single-celled microspores are haploid cells that can develop into double haploid plants and have been widely used as a breeding tool to generate homozygous plants within a generation. In this study, we combined the CRISPR/Cas9 system with microspore technology and developed an optimized haploid mutagenesis system to induce genetic modifications in the wheat genome. We investigated a number of factors that may affect the delivery of CRISPR/Cas9 reagents into microspores and found that electroporation of a minimum of 75,000 cells using 10–20 µg DNA and a pulsing voltage of 500 V is optimal for microspore transfection using the Neon transfection system. Using multiple Cas9 and sgRNA constructs, we present evidence for the seamless introduction of targeted modifications in an exogenous *DsRed* gene and two endogenous wheat genes, including *TaLox2* and *TaUbiL1*. This study demonstrates the value and feasibility of combining microspore technology and CRISPR/Cas9-based gene editing for trait discovery and improvement in plants.

## Introduction

Wheat is a primary staple food crop providing 20% of the calorie and protein intake for the global population. In recent years, wheat production increasing at less than 1% per year, has lagged behind the demands from a rising global population. Improving wheat yield without compromising grain quality is a complex challenge that will require development and adoption of novel genome analysis and precision editing technologies. Recent advances in double haploid (DH) technology have provided a strategy to speed up wheat breeding and biotechnology^1^.

Gene editing based on a bacterial adaptive immune system, termed CRISPR/Cas9 (Clustered, Regularly Interspersed, Palindromic Repeats/CRISPR-associated endonuclease 9)^2–5^ has sparked a new revolution in biological and agricultural research^6,7^. CRISPR/Cas9 can generate targeted gene knock-outs and replacements which are invaluable for understanding the function of genes. Additionally, this innovative technology offers an efficient approach for genetic manipulation of crops without the retention of large transgene sequences in the final plant variety^8–10^. A variety of tools have been developed to optimize the components of the CRISPR/Cas9 system. We have recently developed a web-based bioinformatics tool, WheatCRISPR (https://crispr.bioinfo.nrc.ca/WheatCrispr/), to design specific gRNAs for genome editing and transcriptional regulation of gene expression in wheat (Cram *et al*., submitted). Although CRISPR/Cas9 has been demonstrated in wheat^11–15^, deployment of this technology in crop development is limited by the amount of time and resources required to produce modified homozygous genotypes through the conventional plant transformation based gene editing methods. Transformation based approaches are laborious and time consuming as they require multiple generations. In addition, only some varieties of wheat (e.g., Fielder and Bobwhite) can be easily transformed which limits applications of CRISPR in wheat.

Microspores, or immature pollen grains, are haploid cells that have the ability to regenerate into whole plants when exposed to appropriate culture conditions and stimuli. Cellular totipotency of microspores has been routinely exploited in the generation of doubled haploid populations for crop research and breeding^16^. A functional microspore-based gene editing system can offer several advantages compared to conventional plant transformation-based systems. For example, a large number of genetically identical and physiologically uniform embryogenic microspores can be easily isolated in a relatively short time and used as explants for transformation. After transformation, the microspores are screened for gene editing events and then homozygous plants are generated after diploidization. In contrast, it takes a significantly higher level of effort, labor and time to prepare a similar number of immature embryos as targets for genetic transformation. Additionally, transgenic plants regenerated from transformed immature embryos are usually heterozygous or hemizygous for the transgene and homozygous transformants can be selected only in the next generation. The microspore-based haploid strategy also reduces the number of alleles to be edited by half; in hexaploid wheat, the number of alleles to be edited is reduced from six to three. Thus, the relative ease of isolation and the potential for generation of homozygous diploid transgenic plants in one step makes single cell microspores a highly desirable target for genome editing^17–19^, particularly in transformation-recalcitrant species such as wheat with long generation times.

In this study, we combined microspore technology and CRISPR/Cas9 gene editing to facilitate the efficient and widespread use of CRISPR/Cas9 technology in haploid systems. We report optimised methods for delivery of CRISPR/Cas9 reagents into wheat haploid cells using the Neon electroporation system (Thermo Fisher Scientific), followed by regeneration of transformed microspores into doubled haploid transgenic plants. Furthermore, we successfully demonstrate the potential of microspores as explants for CRISPR/Cas9-based haploid mutagenesis by inducing targeted modifications in an exogenous *DsRed* gene and two endogenous wheat genes, including *TaLox2* and *TaUbiL1*.

## Results and Discussion

### CRISPR/Cas9 delivery: microspore transfection using the Neon electroporation system

A reliable and efficient foreign DNA delivery system is essential for microspore-based gene editing. Microspore transformation could be performed by electroporation, particle bombardment or by co-cultivation of androgenic microspores with *Agrobacterium tumefaciens*. Electroporation-based transfection is relatively simple, less cumbersome and more efficient. It has been used for DNA delivery into protoplasts and microspores^17,20,21^. The Neon transfection system developed by Thermo Fisher Scientific offers a streamlined electroporation process for fast and efficient delivery of nucleic acids into mammalian cells^22^. In contrast to other cuvette-based electroporators, such as Gene-Pulser (Bio-Rad, USA), ECM (BTX, USA) or NEPA21, the Neon system uses a biologically compatible pipette tip chamber that generates a more uniform electric field and has been reported to allow better maintenance of physiological conditions resulting in very high rates of cell survival. Although the Neon system is widely used for mammalian cell transfection, it had not been previously applied to plant cell transformation.

In this study, to test the feasibility of using the Neon transfection system for plant transformation, we evaluated the efficacy of delivery of Cas9 into microspores of the wheat cultivar AC Nanda. A plasmid (pPZP202_DsRed_Cas9)^23^ comprising the *Streptococcus pyogenes* Cas9 and two selection markers was used for transformation. The selection markers were chosen to help assess the efficiency of transfection and included a red fluorescent protein variant DsRed and hygromycin phosphotransferase. The electroporation experiments involved the counting of the DsRed expressing viable microspores stained with fluorescein diacetate in response to different parameters such as pulsing voltage, the composition of the electroporation buffer, number of microspores and the amount of plasmid DNA.

Cell survival and transfection efficiency (TE) are greatly affected by pulsing field strength (ie., pulse voltage, width, and number), electroporation buffer composition, DNA concentration and the number of cells. For microspore electroporation, we initially followed Thermo Fisher’s standard recommendations for pulse strength (ie., 3 pulses of 1400 V, 30 seconds each), and a dense microspore population (200,000) and DNA concentration (30 µg). The initial analyses were unsuccessful as we could not detect the expression of DsRed. The higher strength of pulsing voltage originally optimized in animal models was found to be lethal to plant microspores as most of the cells did not survive post-electroporation at 1400 V. This indicated a need for optimization of electroporation parameters.

**Figure 1:**
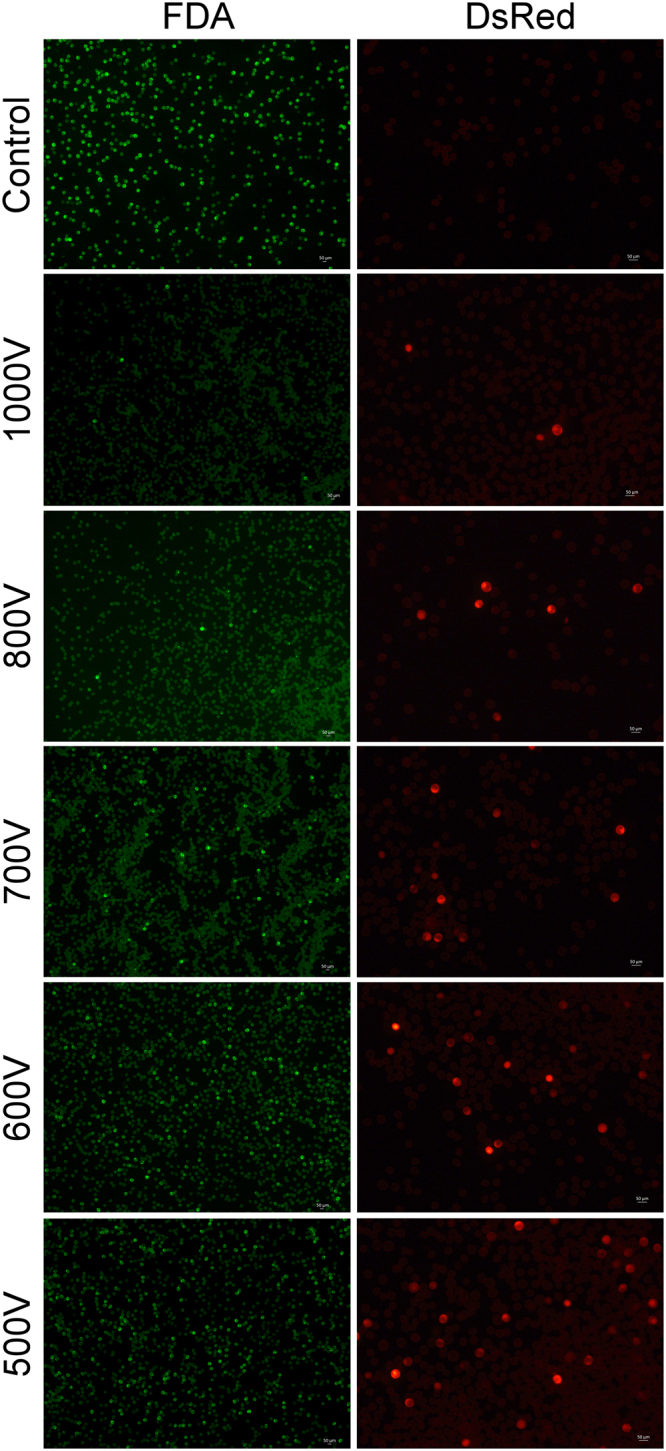
Effect of pulsing voltage on delivery of Cas9 and DsRed expression construct into wheat microspores using the Neon electroporation system. Representative images of fluorescein diacetate (FDA) stained viable and DsRed expressing wheat microspores after 48 h after electroporation are shown. The highest microspore survival and transfection efficiency were observed at 500 V (1.6% transfection efficiency, 50% cell viability).

The optimization of the voltage is an important step in the development of a transfection protocol by electroporation. The voltage must be high enough to create pores in the cell membrane, yet it must be low enough to avoid excessive cell death. We evaluated the effect of a wide range of pulsing voltages (500 to 1400 V; 500 V is the lower limit of the input voltage in the Neon transfection system) on microspore TE and survival. As shown in Fig. 1, the microspore viability and TE increased with a decrease in pulsing voltage and the highest microspore survival and TE was observed at 500 V (1.6% TE with 50% cell viability). TE was calculated as a function of the number of viable microspores expressing DsRed after electroporation.

TE (%) = (number of DsRed expressing microspores/total number of microspores) × 100.

**Figure 2:**
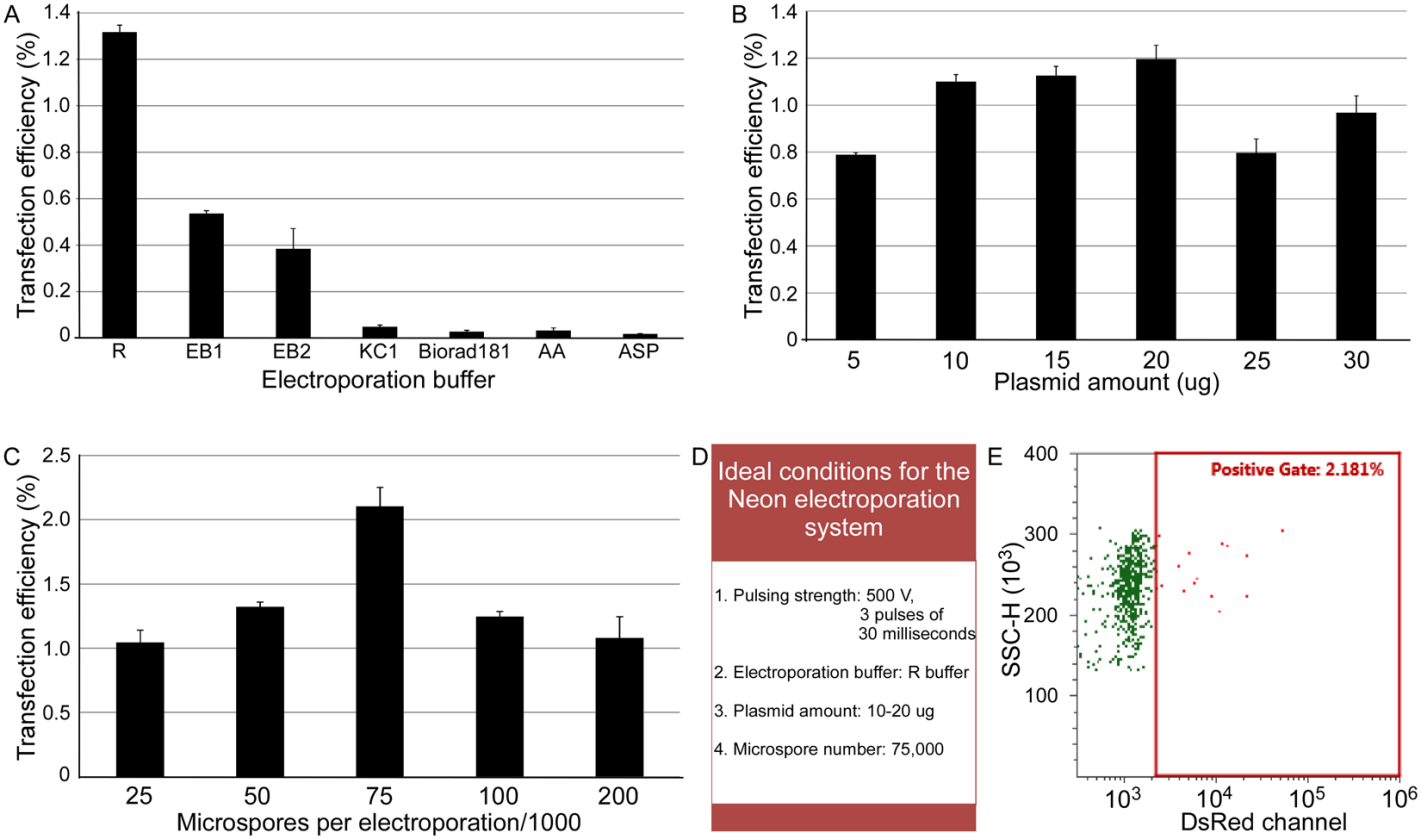
Optimization of electroporation parameters for the delivery of Cas9 and DsRed expression construct into wheat microspores. The effect of buffer composition (**A**), DNA concentration (**B**) and microspore density (**C**) on transfection efficiency is shown. The microspore transfection using optimised electroporation parameters (**D**) resulted in the highest transfection efficiency of 2.2% (**E**), as measured by flow cytometry. The error bars represent standard error of the mean (SEM).

The chemical composition of the electroporation buffer also plays an important role in transfection by electroporation. We evaluated the effect of six different buffer compositions reported in the literature^24^ on TE and compared with the Neon Resuspension buffer (R buffer). The electroporation in the R buffer resulted in the highest transfection efficiency (1.3%) followed by the buffer EB1 (0.53%) and EB2 (0.38%), while the other four buffers gave a significantly lower transfection efficiency (Fig. 2A). The R buffer is an organic acid based buffer. Because of the low cell toxicity, the organic acid based buffers are more suitable than buffers with chlorides^24^.

Wheat microspore TE was further optimized using different amounts of the plasmid DNA starting from 5 to 30 µg. The highest TE of 1.2 per cent was observed with 20 µg of plasmid DNA, however, the increase in TE was not significant compared to the TE with 10 or 15 µg of plasmid DNA (Fig. 2B). A number of previous reports^17,25^ had recommended 100 to 200 µg of DNA for successful wheat and maize microspore electroporation. But in our study plasmid DNA higher than 20 µg (25 and 30 µg) did not increase the number of DsRed-expressing microspores, indicating a saturation level at 20 µg (Fig. 2B).

We further assessed the effect of cell density on TE, while keeping the voltage (500 V), buffer (R buffer) and DNA concentration (20 µg) constant (Fig. 2C). The number of DsRed expressing microspores increased in correlation to the microspore density (Fig. 2C) with the TE maximised (2.12%) when 75,000 microspores were used for electroporation. Increasing the number of microspores higher than 75,000 was counterproductive, as it resulted in a decreased number of DsRed expressing microspores (Fig. 2C).

**Figure 3:**
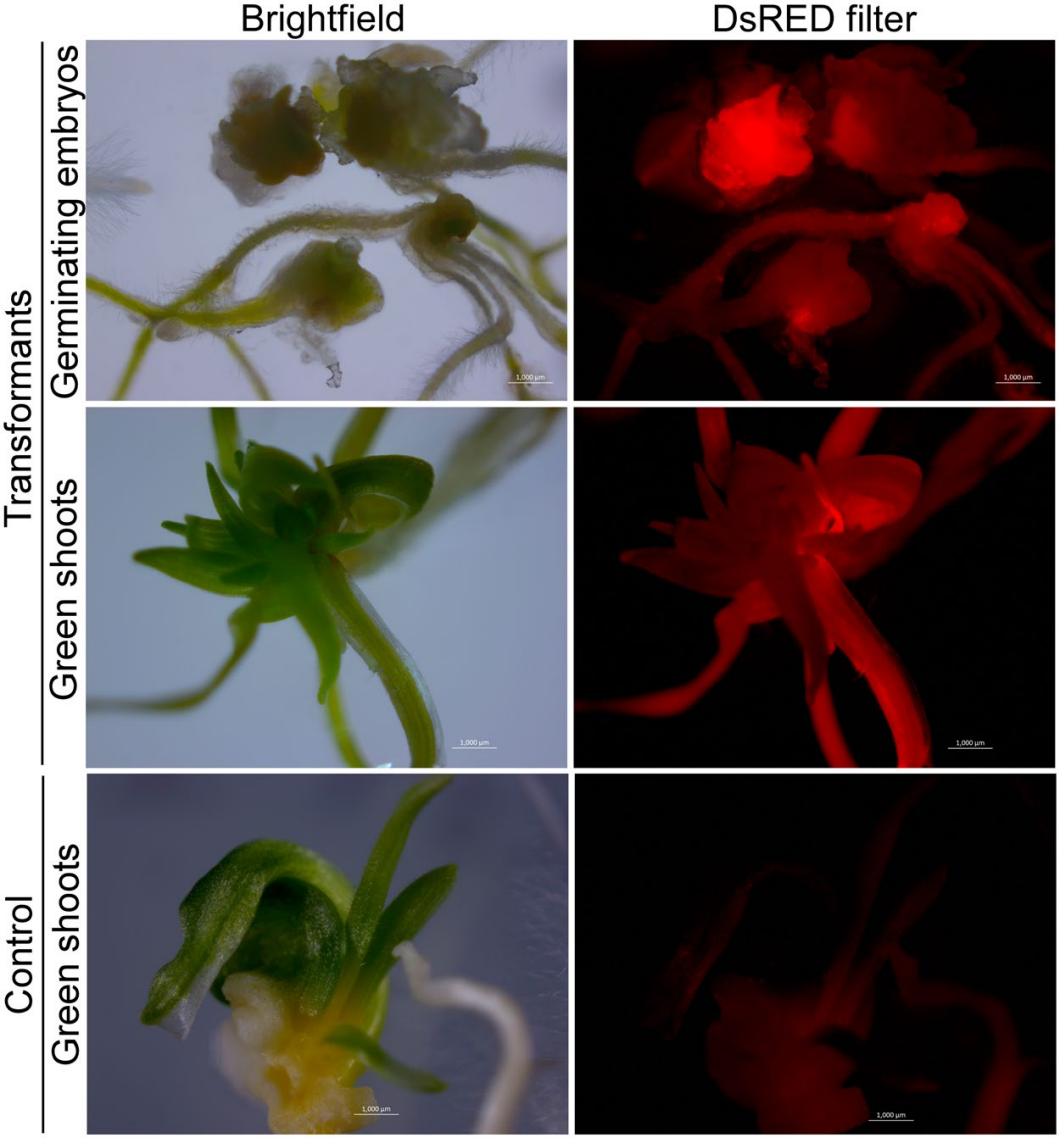
Microspore-derived homozygous doubled haploid plants. Epifluorescent microscopy images showing germinating embryos and green shoots regenerated from wild type (control) and transformed microspores (transformants) expressing DsRed. Scale bar, 1000 µM.

In summary, the optimised protocol for wheat microspore transfection using the Neon system involves electroporation of a minimum of 75000 cells in the R buffer using 10–20 µg of foreign DNA at the pulse strength of 500 V (3 pulses of 30 seconds each; Fig. 2D). Microspore transfection using these optimised electroporation parameters resulted in the highest TE of 2.2% (Fig. 2E). Electroporation of microspores with the Neon system did not affect their regenerative potential, as they were able to produce DsRed expressing plantlets (Fig. 3) after regeneration using standard microspore culture protocols^26^.

### CRISPR/Cas9-based targeted mutagenesis of an exogenous *DsRed* reporter gene in wheat microspores

Having optimised the methods for delivery of DNA and regeneration of microspores through embryogenesis, we set out to examine if microspores can be used for gene editing. To achieve this, we selected the pPZP202_DsRed_Cas9 plasmid, containing the *DsRed* reporter gene as a target of CRISPR/Cas9-mediated mutagenesis, and pZK_gDsRed-2, a previously validated sgRNA expression cassette successful in targeting *DsRed*^23^. Successful targeting of the *DsRed* reporter gene by gDsRed-2/Cas9 in wheat microspores is expected to introduce a double stranded break at the target site in *DsRed* which is repaired through non-homologous end joining (NHEJ) leading to mutations (insertions/deletions), some of which may be frameshift mutations resulting in the abolition of the ability of DsRed to produce a fluorescent signal.

**Figure 4:**
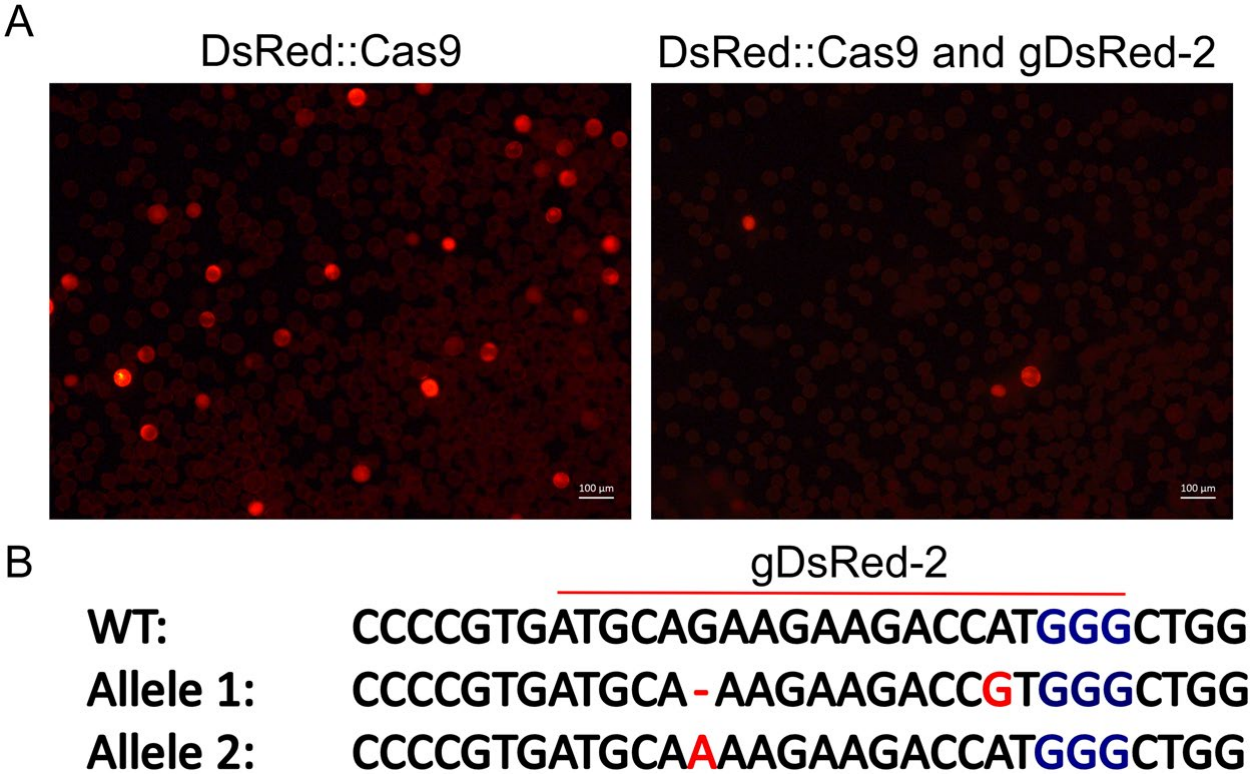
CRISPR/Cas9-based editing of the exogenous *DsRed* reporter gene in wheat microspores. (**A**)Fluorescence emitted from DsRed produced in microspores co-transfected with the DsRed_Cas9 and gDsRed-2 constructs (right panel) compared to control cells expressing only DsRed_Cas9 construct (Left panel). (**B**)Mutations detected at or near the cleavage site in sequenced products of *DsRed* from microspores co-transfected with the DsRed_Cas9 and gDsRed-2 constructs.

The plasmid DNA of pPZP202_DsRed_Cas9 and pZK_gDsRed-2 (Fig. S1) was co-transfected into wheat microspores using the Neon electroporation system. As a control, microspores were transfected with only pPZP202_DsRed_Cas9. After 48 h, the transfected microspores were examined for the presence of DsRed by fluorescence microscopy. While the viability of control and co-transfected microspores was comparable (~50% in both cases), a significant reduction (92%) in the number of microspores expressing DsRed was observed in cells co-expressing the DsRed_Cas9 and gDsRed-2 compared to control cells expressing only DsRed_Cas9 (Fig. 4A), suggesting successful disruption of the open reading frame (ORF) of the target *DsRed* gene by gDsRed-2/Cas9. To confirm frameshift mutations in *DsRed* ORF, total DNA from pooled microspores was isolated, and a 570 bp region encompassing the target site was PCR-amplified, cloned into an Invitrogen TOPO-Blunt plasmid and 96 clones were sequenced by Sanger sequencing. Two single bp substitutions and a deletion were detected at or near the cleavage site in 2 out of the 96 sequenced products (Fig. 4B). These results demonstrated that CRISPR/Cas9 system can be used to induce targeted mutagenesis in wheat microspores.

### CRISPR/Cas9-based targeted mutagenesis of endogenous genes in wheat microspores

**Figure 5:**
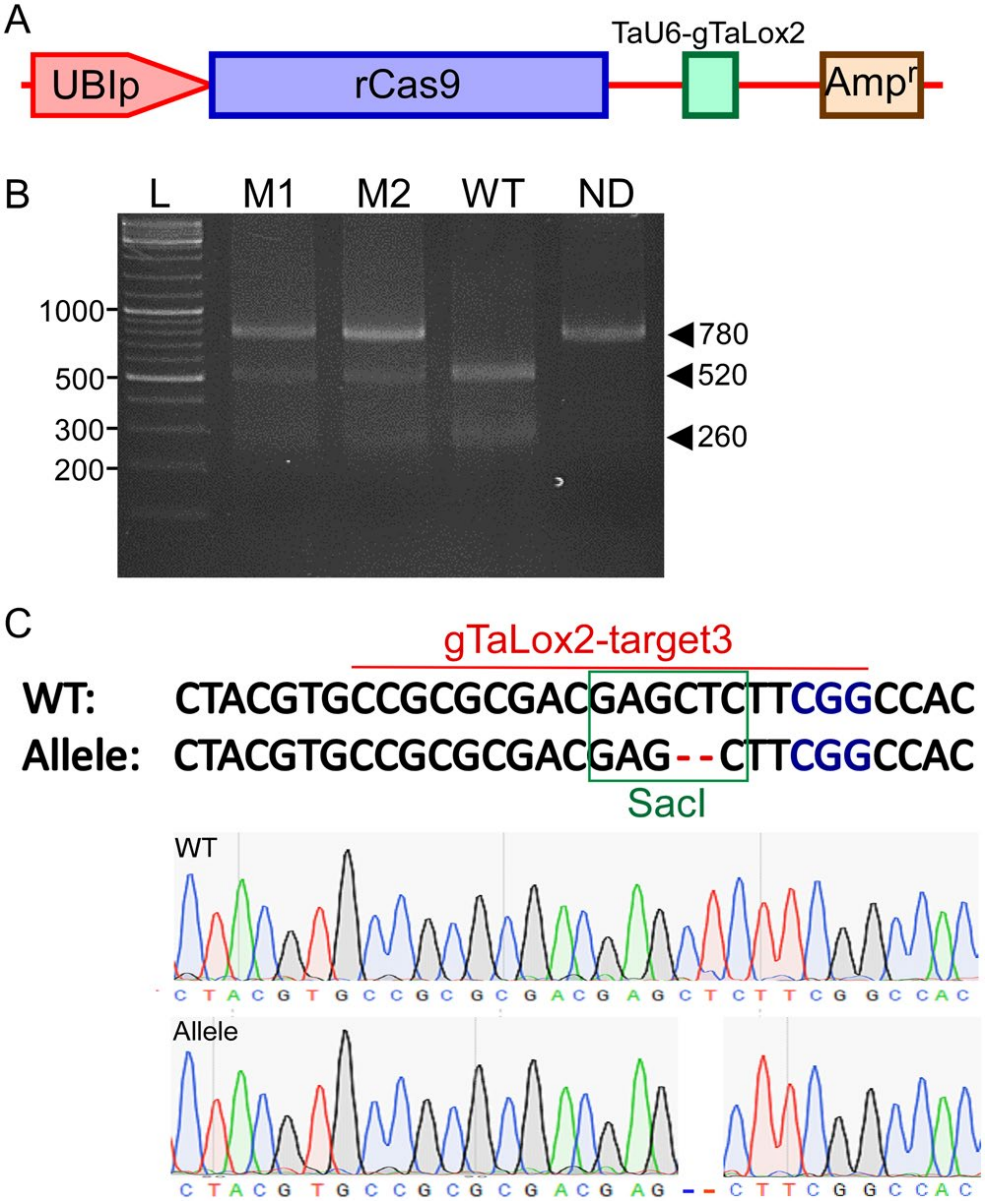
CRISPR/Cas9-based editing of the endogenous *TaLox2* gene in wheat microspores. (**A**) Schematic diagram showing the pJIT163-2NLSCas9/gTaLox2-target3 construct used for mutagenesis of *TaLox2* gene in wheat microspores. (**B**) PCR/restriction enzyme assay to detect mutations in the *TaLox2* gene induced by Cas9/ gTaLox2. (**C**) A 2 bp deletion detected at the cleavage site in the sequenced product of *TaLox2* from microspores transfected with pJIT163-2NLSCas9/gTaLox2-target3 construct. Sanger sequencing electropherograms are shown below.

To further test the effectiveness of the CRISPR/Cas9 system in editing endogenous genomic loci in microspores, two wheat genes *TaLox2* and *TaUbiL1* were chosen. TaLox2 encodes a lipoxygenase enzyme involved in the hydrolysis of polyunsaturated fatty acids, such as linoleic acid (LA), α-linolenic acid and arachidonic acid^27^. A sgRNA designed to target the fourth exon of *TaLox2* (gTaLox2-target3) was previously shown to induce mutations in wheat protoplasts^28^. The pJIT163-2NLSCas9/gTaLox2-target3 plasmid (Fig. 5A) designed to express this sgRNA under the control of the TaU6 promoter and the Cas9 was transfected into wheat microspores. The Cas9/gTaLox2-target3 cleavage site coincides with the SacI restriction enzyme recognition sequence; thus successful cleavage and an NHEJ-mediated frameshift mutation at the target site are expected to disrupt restriction enzyme digestion. Total DNA from the pool of transfected microspores was isolated about 48 h after transfection. A 780 bp region encompassing the cleavage site was PCR-amplified and digested with the SacI restriction enzyme. Agarose gel electrophoresis of digested products revealed two bands at the expected sizes of 520 and 260 bps in the wild-type microspore DNA sample, whereas, the DNA sample from the microspores expressing pJIT163-2NLSCas9/gTaLox2-target3 exhibited an additional band at 780 bp that is identical in size to the undigested product (Fig. 5B). Gel-purification and Sanger sequencing of the undigested band revealed the presence of a small 2 bp deletion at the predicted cleavage site (Fig. 5C), suggesting the successful introduction of a double strand break by Cas9/gTaLox2-target3 followed by erroneous DNA repair via the NHEJ pathway.

**Figure 6:**
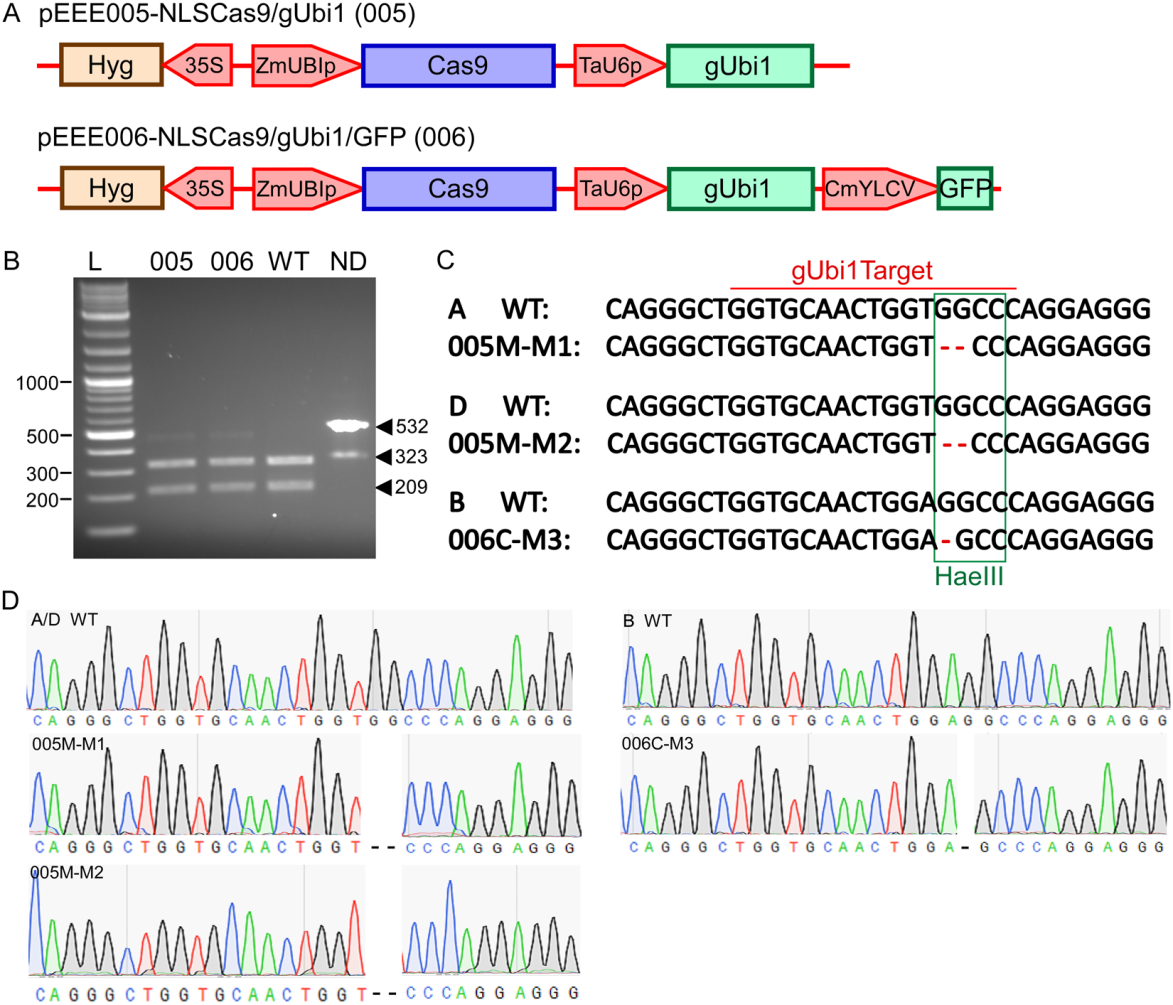
CRISPR/Cas9-based editing of an endogenous *ubiquitin* gene in wheat microspores. (**A**) Schematic diagram of the pEEE005-NLSCas9/gUbi1 and pEEE006-NLSCas9/gUbi1/GFP constructs used for mutagenesis of in wheat microspores. (**B**) PCR/restriction enzyme assay to detect mutations in *TaUbiL1* induced by Cas9/ gUbi1. (**C**) Deletions detected at the cleavage site in sequenced products of all three homoeologs of *TaUbiL1* from wheat microspores transfected with pEEE005-NLSCas9/gUbi1 or pEEE006-NLSCas9/gUbi1/GFP constructs. The three homoeologues of *TaUbiL1* were defined based on multiple single nucleotide polymorphisms in each background (sequence not shown) for the three genomes in this locus. (**D**) Sanger sequencing electropherograms showing mutations in *TaUbiL1* homoeologues from the **A**, **B** and **D** genomes of wheat.

A sgRNA designed to target the third exon of a *ubiquitin* gene *TaUbiL1* was previously shown to induce mutations in wheat protoplasts and scutella^29^. The pEEE005-NLSCas9/gUbi1 and pEEE006-NLSCas9/gUbi1/GFP plasmids (Fig. 6A) expressing the sgRNA from the control of the TaU6 promoter were transfected into wheat microspores. The Cas9/gUbi1 cleavage site lies at the same location as the HaeIII restriction enzyme recognition sequence. As with the *TaLox2* locus, successful NHEJ-mediated frameshift mutations should prevent restriction enzyme digestion. Total DNA from the pool of transfected microspores was isolated about 48 hr after transfection. A 532 bp region containing the target site was PCR-amplified and purified by gel extraction. Amplicons were then digested with a HaeIII restriction enzyme. Agarose gel electrophoresis of digested products revealed two bands at expected sizes of 323 bp and 209 bp. The DNA from edited microspores, pEEE005 and pEEE006, contained an additional band at 532 bp that is identical to the undigested product (Fig. 6B). Gel-purification and Sanger sequencing of the undigested band revealed the presence of multiple insertions and deletions at the predicted cleavage site in all three homoeologues (A, B and D subgenomes) of the *Ubiquitin* gene (Fig. 6C) suggesting the successful introduction of double strand breaks followed by erroneous DNA repair via NHEJ pathway.

### Concluding remarks

CRISPR-Cas9 technology has become a promising technique for trait discovery and improvement in plants. Plant haploid germ cells are a valuable resource for reverse genetic approaches and their mutagenesis using the CRISPR/Cas9 system can enable efficient manipulation and functional validation of genes. In this study, we successfully evaluated the feasibility of inducing genetic modifications in wheat using microspores as explants. The standardized protocols for the delivery of CRISPR/Cas9 components into microspores using the Neon electroporation system and detection of genetic modifications in exogenous and endogenous genes described in this study provide an efficient alternative to the laborious and time-consuming traditional somatic cell transformation-based editing of the wheat genome. The Neon electroporation method resulted in a TE of 2.2% (~4.5% if only viable cells were considered). We expect further improvements to TE with a progressive decrease in pulsing voltage beyond 500 V, which is the lower limit of input voltage of the currently available Neon transfection system.

As demonstrated in this study and previously^17,26,30–32^, well-established protocols exist for regeneration of microspores; however, the regeneration efficiency is still much lower (100 to 700 green plantlets from 100,000 microspores). The genotype of the donor plant and growing conditions can have a significant impact on regeneration efficiency. Additionally, lower transfection and gene editing efficiencies can have a further negative effect on successful regeneration of genetically modified plants. It is advisable to minimise the background of non-transfected cells by incorporating a non-destructive, easily detectable fluorescence protein marker on gene editing constructs. Fluorescence activated cell sorting (FACS) system can then be applied to enrich transfected cells. Also to improve the chances of regenerating successfully edited plants, designing highly specific and effective gRNAs is important. Multiple bioinformatics tools, such as WheatCRISPR (Cram *et al*., submitted), E-CRISP^33^ and CRISPRdirect^34^, have been developed to facilitate the design of gRNAs targeting specific loci in the wheat genome and prediction of off-target sites. Among these, the WheatCRISPR tool offers a distinct advantage of selection of effective gRNAs based on their predicted high on-target and low off-target activity scores, calculated using Doench algorithms^35^, as well as other characteristics such as position within the targeted gene. Recently, DNA-free genome editing with direct delivery of preassembled CRISPR/Cas9 ribonucleoprotein complex was successfully used to achieve targeted mutagenesis in the protoplasts derived from *Arabidopsis thaliana*, tobacco, and lettuce as well as in rice plants^36^. Genome-editing using DNA free systems could circumvent restrictive GMO regulations, thus paving a way for widespread use of this innovative technology in trait discovery and improvement. Microspores provide a powerful alternative to protoplast culture due to their relatively higher and synchronised regeneration potential. An important next step would be to evaluate and optimise an efficient transfection system to directly deliver CRISPR/Cas9 ribonucleoproteins into microspores and assess the editing efficiency.

In summary, the microspore-based gene editing system described in this study provides an innovative platform for functional validation of genes as well as genetic characterization and improvement of valuable traits. This system, in combination with fluorescent reporter systems, can also be used for high throughput screening of multiple gRNAs simultaneously. As an elegant alternative to reliance on traditional somatic cell transformation-based gene editing, microspore-based gene editing system has the potential to accelerate the pace of gene discovery and crop breeding.

## Materials and Methods

### Plant material and microspore extraction

Donor plants of the spring wheat cultivars Bobwhite (a cultivar from CIMMYT) and AC Nanda^37^, which are highly responsive to stress-induced microspore embryogenesis, were used for microspore extraction. Seeds of Bobwhite and AC Nanda were obtained from Plant Gene Resources of Canada and Agriculture and Agri-Food Canada, respectively. The method of isolation of wheat microspores from cold-treated spikelets was adopted from Eudes and Amundsen (2005). Prior to microspore extraction, tillers were harvested from plants before the spike emerged from the boot and kept in the dark at 4 °C for 21+/−3 days in distilled water. The spikes were removed from the boot, sterilized, and anthers were excised and placed into Eudes extraction buffer. Anthers were macerated using Warring blender cup (VWR international, #58983-093) strained through a 70 micron filter (VWR International, #CA21008-950), and washed several times with Eudes extraction buffer^26^ by centrifugation using a swinging bucket rotor. Finally, cells were re-suspended in NPB99^26^ liquid media and used for electroporation.

### Assembly of pEEE005-NLSCas9/gUbi1 and pEEE006-NLSCas9/gUbi1/GFP

Vectors were assembled by first hybridizing and annealing gRNA oligos oEE124 5′-ACTTGCTGGTGCAACTGGTGGCCCG-3′and oEE125 5′-AAAACGGGCCACCAGTTGCA CCAGC-3′. They were then cloned into pJG310 vector using the Esp31 enzyme and a Golden Gate procedure. Final assembly into pEEE005 (Fig. S2) and pEEE006 (Fig. S3) was performed using the Golden Gate assembly toolkit described by Čermák *et al*.^38^. Individual module vectors were pTRANS_210, pMOD_A1110, and either pMOD_C0000 for pEEE005 or pMOD_C3003 for pEEE006.

### Microspore electroporation

Isolated microspores were pelleted by spinning them at 1000 rpm for three minutes in a benchtop microcentrifuge, in aliquots of 25,000 to 200,000 cells per 1.5 mL Eppendorf tube as required for the individual treatment. The NPB99 media was then removed, and the cells were re-suspended in 100 uL of the electroporation buffer being used for the experiment. Depending on the treatment, 5 to 30 ug of plasmid was added to each tube containing cells and DNA were mixed by gently flicking the tube.

Electroporation was then carried out as described in the Neon (Neon Transfection System MPK5000) instruction manual in 100 µL volumes. Multiple parameters affecting TE, including pulsing voltage, buffer composition, amount of DNA and microspore number, were tested as described in the results section. At least three biological replicates for each treatment were electroporated and plated for observation and regeneration.

### Evaluation of microspore viability and transfection efficiency

Evaluation of microspore viability was performed before and after electroporation by staining with 0.01% fluorescein diacetate and observing under a Zeiss Axio Zoom V16 stereo microscope. Forty-eight hours after transfection, images were acquired and transfection efficiency was calculated by comparing the number of DsRed positive cells (detected with a 43HE DsRed filter-excitation wavelength 538–562 nm and emission wavelength 570–640 nm) to the total number of microspores.

### Regeneration of transformants from electroporated microspores

Immediately after electroporation, microspores were plated on glass bottom Petri dishes containing 3 mL of NPB99 liquid media and sealed with Parafilm. Microspores were co-cultured with living mature ovaries (3–4 ovaries per plate) at 28 °C in the dark for embryoid development. After 20–30 days, embryos larger than 0.5 mm were removed from the Petri dishes and plated onto GEM culture medium^39^. The petri dishes were sealed with parafilm and placed 30 cm beneath Sylvania Pentron 4100 K spectrum bulbs (21 watts) delivering 125 µmol m^−2^s^−1^ lights (16 h light period) at 25 °C constant temperature. Once the embryos turned green, they were aseptically transferred onto 50 ml rooting media in magenta vessels under the same conditions.

### Confirmation of DNA delivery and mutation detection using PCR/Restriction Enzyme assay

In order to confirm plasmid DNA delivery, genomic DNA was extracted from pooled putative transformed microspores. The targeted *DsRed* and *TaLOX2* gene fragments were then amplified using gene specific primers: DsRed-1F: 5′-AGTTCCAGTACGGCTCCAAGGTGTACGT-3′; DsRed-1R: 5′-TCGGTGCGCTCGTACTGCTCCACGAT-3′; TaLOX-1F:5′-GTGCCGCGCGACGAGCTCT T-3′; TaLOX-1R: 5′-AAACAAGAGCTCGTCGCGCGGCA-3′ (Figs 4 and 5).

All PCR fragments were purified using a QIAGEN kit and were digested with restriction enzymes assuming that if an edit had occurred and NHEJ erroneously repaired the cut, the restriction site should have been destroyed. The length of the *TaLox2* PCR product was 780 bp, and the complete SacI digestion of PCR product from the wild-type gDNA gave only two bands (520 bp and 260 bp) and the mutant gDNA had three bands (260 bp, 520 bp, and 780 bp). Therefore, the undigested 780 bp band which corresponded to mutant gDNA was gel extracted and cloned into a TOPO-Blunt plasmid and sequenced for mutation detection.

The targeted *ubiquitin* gene fragment was amplified using Ubi Gene specific Primers: Ubi1 F:5′-CAAACCCCTGGAGCAA-3′; Ubi1 R:5′-CACAGTTGCTTAGCATGAACC-3′. The length of the Ubi1 PCR product was 532 bp, and complete HaeIII digestion of PCR products from wild-type gDNA produced two cut bands at 323 bp and 209 bp while the mutated samples had three bands (209 bp, 323 bp, and 532 bp). The undigested, full-length, bands in the mutant samples were gel extracted and cloned into cloning vector pJet for Sanger sequencing.

### Data availability statement

All data generated or analysed during this study are included in this published article. Vector constructs and other materials described in the study will be made available upon request.

## Electronic supplementary material

Electronic Supplemental Material
