# Supplementary material for: Targeted mutagenesis in wheat microspores using CRISPR/Cas9
Source: Sci Rep. 2018 Apr 25;8:6502. doi: 10.1038/s41598-018-24690-8 (PMC5916876; doi:10.1038/s41598-018-24690-8)
Supplement: Supplementary file 1 — Electronic Supplemental Material [file 41598_2018_24690_MOESM1_ESM.docx]

**Targeted mutagenesis in wheat microspores using CRISPR/Cas9**

Pankaj Bhowmik^1*^, Evan Ellison^2^, Brittany Polley^1^, Venkatesh Bollina^1^, Manoj Kulkarni^1^, Kaveh Ghanbarnia^1^, Halim Song^1^, Caixia Gao^3^, Daniel F. Voytas^2^, Sateesh Kagale^1*^

^1^Canadian Wheat Improvement Flagship Program, National Research Council Canada, 110 Gymnasium Place, Saskatoon, SK, S7N 0W9, Canada

^2^Department of Genetics, Cell Biology, and Development, Center for Genome Engineering, University of Minnesota, Saint Paul, MN 55108, USA

^3^State Key Laboratory of Plant Cell and Chromosome Engineering, Institute of Genetics and Developmental Biology, Chinese Academy of Sciences, Beijing 100101, China

*Correspondence and requests for materials should be addressed to P.B. (email: [Pankaj.Bhowmik@nrc-cnrc.gc.ca](file:///C:\Users\kagales\Desktop\Work\Manuscripts\Neon%20Electroporation%20-%20Pankaj\Manuscript%20v1\Pankaj.Bhowmik@nrc-cnrc.gc.ca)) or S.K. (email: [Sateesh.Kagale@nrc-cnrc.gc.ca](file:///C:\Users\kagales\Desktop\Work\Manuscripts\Neon%20Electroporation%20-%20Pankaj\Manuscript%20v1\Sateesh.Kagale@nrc-cnrc.gc.ca))

**Electronic Supplemental Material
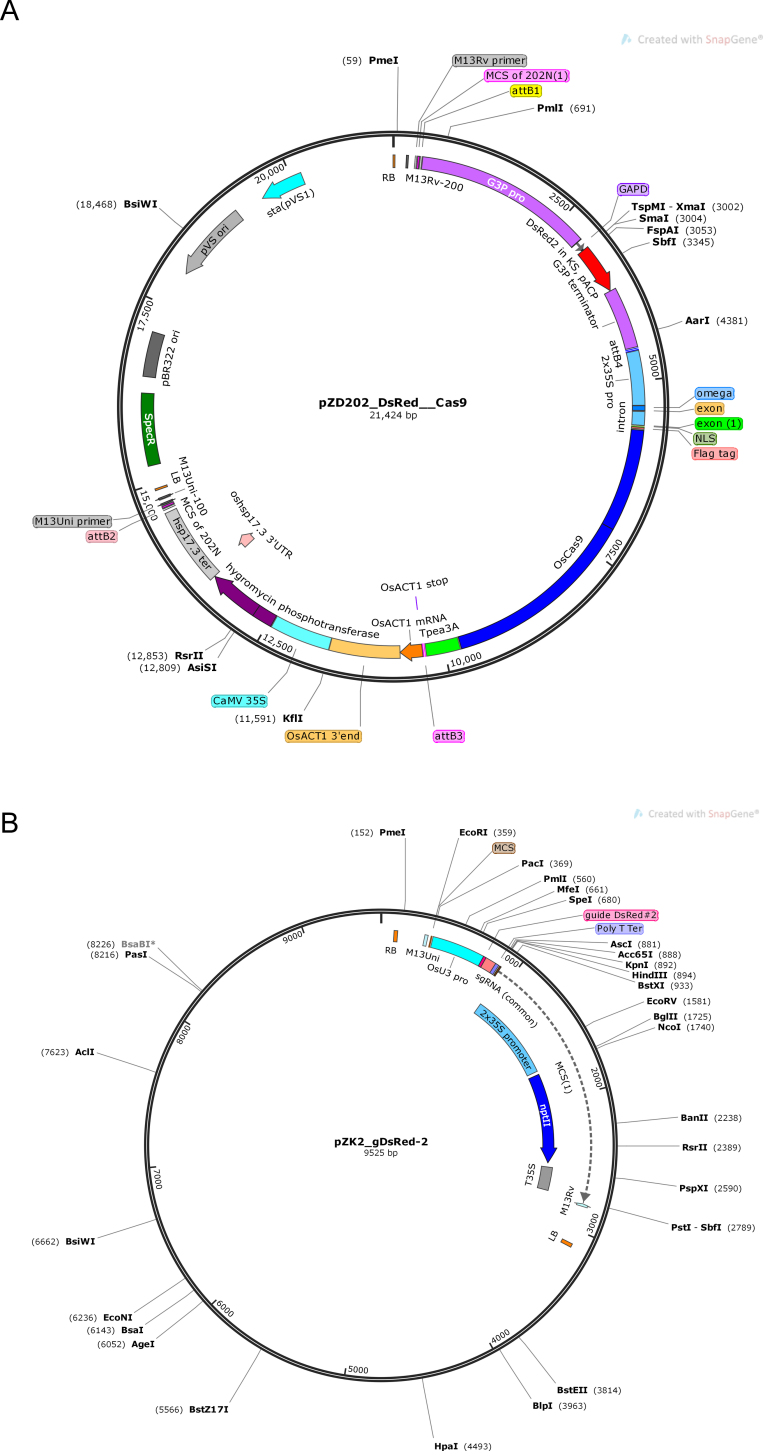
**

**Figure S1. Schematic illustration of the pPZP202_DsRed_Cas9 and pZK_gDsRed-2 vectors (Mikami et al., 2015).**

**
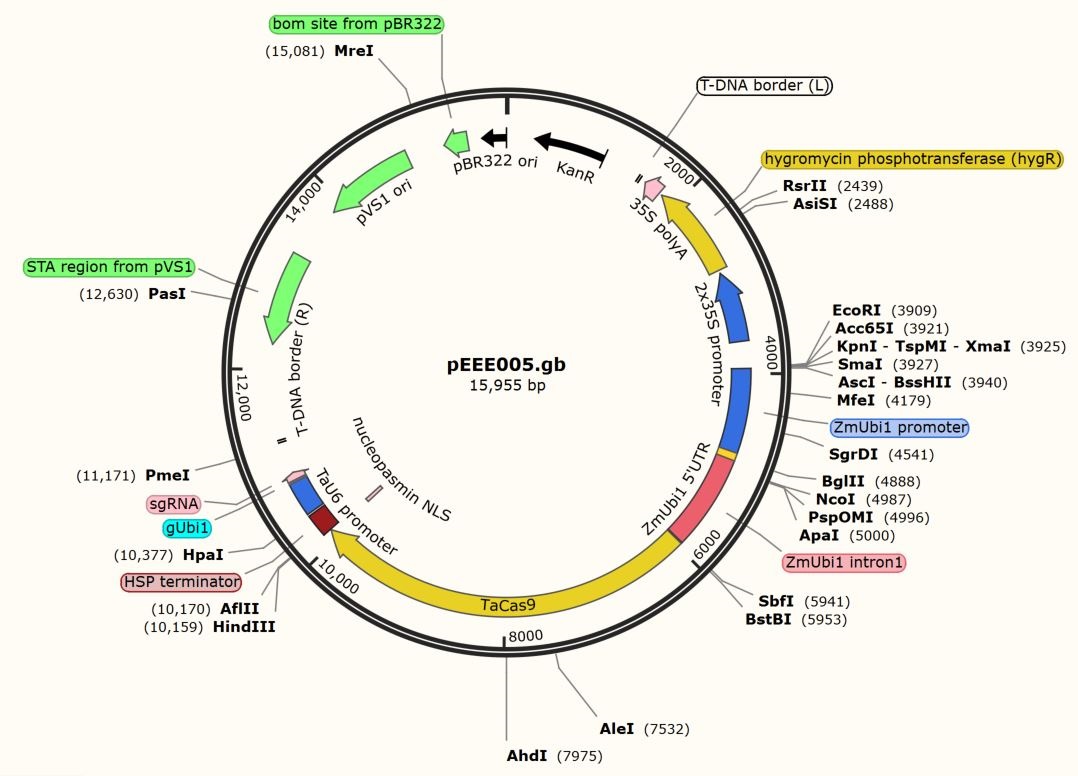
**

**Figure S2. Schematic illustration of the pEEE005 vector.**

**
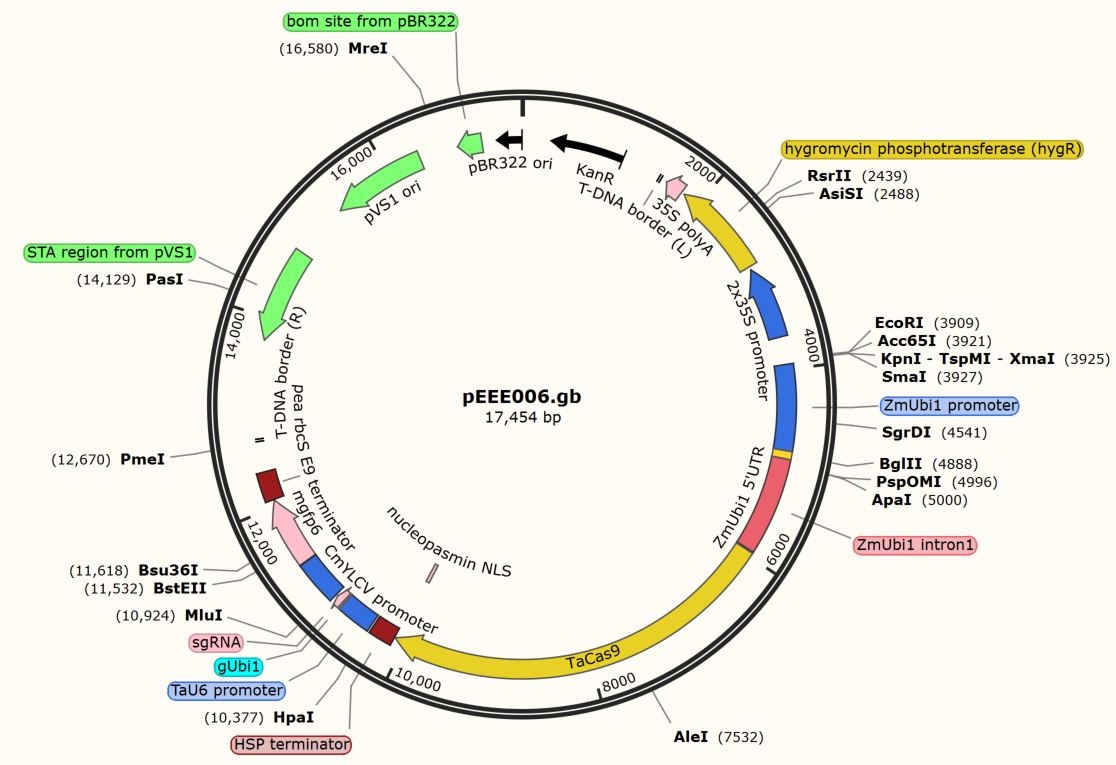
**

**Figure S3. Schematic illustration of the pEEE006 vector.**
